# Molecular Screening and Characterization of Enteric Protozoan Parasites and Microsporidia in Wild Ducks from Portugal

**DOI:** 10.3390/ani14202956

**Published:** 2024-10-14

**Authors:** Sara Gomes-Gonçalves, David Rodrigues, Nuno Santos, Nausicaa Gantois, Magali Chabé, Eric Viscogliosi, João R. Mesquita

**Affiliations:** 1 School of Medicine and Biomedical Sciences, Porto University, 4050-313 Porto, Portugal; 2 Coimbra College of Agriculture, Polytechnic University of Coimbra, 3045-601 Coimbra, Portugal; 3 Forest Research Centre, School of Agriculture, University of Lisbon, 1649-004 Lisboa, Portugal; 4 Centro de Investigação em Biodiversidade e Recursos Genéticos, InBIO Laboratório Associado, Universidade do Porto, 4485-661 Vairão, Portugal; 5 CNRS, Inserm, CHU Lille, Institut Pasteur de Lille, U1019–UMR 9017–CIIL–Centre d’Infection et d’Immunité de Lille, Université Lille, F-59000Lille, Francemagali.chabe@univ-lille.fr (M.C.); eric.viscogliosi@pasteur-lille.fr (E.V.); 6 Epidemiology Research Unit (EPIUnit), Instituto de Saúde Pública da Universidade do Porto, 4050-600 Porto, Portugal; 7 Laboratório para a Investigação Integrativa e Translacional em Saúde Populacional (ITR), 4050-600 Porto, Portugal

**Keywords:** *Blastocystis* sp., *Cryptosporidium* spp., ducks, molecular epidemiology, zoonosis, migratory waterfowl

## Abstract

**Simple Summary:**

Enteric parasites, including *Blastocystis* sp., *Balantioides coli*, *Cryptosporidium* spp. and microsporidia, are major public health concerns. This study explores the prevalence of these parasites in wild ducks (*Anas acuta*, *Anas platyrhynchos* and *Anas crecca*) in Portugal. It provides the first molecular evidence of *Blastocystis* sp. ST7 and *Cryptosporidium baileyi* in these birds. Both parasites pose zoonotic risks since *Blastocystis* sp. ST7 is linked to human gut microbiota disruption, while *Cryptosporidium* spp. can cause severe gastrointestinal illness, particularly in immunocompromised individuals. This study finds a 2.82% (2/71; 95% confidence interval [CI]: 0.34–9.81) occurrence of both *Blastocystis* sp. and *Cryptosporidium* spp., with no detection of *Balantioides coli* or microsporidia. Migratory ducks, which travel long distances and occupy diverse habitats, can act as vectors for these pathogens, contributing to their spread across regions. These findings emphasize the need for continued monitoring of wild birds and further research on parasite occurrence in waterfowl. Understanding their role in spreading zoonotic diseases is important for public health due to the long travel distances and diverse habitats of migratory species, meaning they can act as carriers of these pathogens, contributing to their spread across regions.

**Abstract:**

Enteric parasites pose significant threats to both human and veterinary health, ranking among the top causes of mortality worldwide. Wild migratory waterfowl, such as ducks, may serve as hosts and vectors for these parasites, facilitating their transmission across ecosystems. This study conducted a molecular screening of enteric parasites in three species of wild ducks of the genus *Anas* (*A. acuta*, *A. platyrhynchos* and *A. crecca*) from Portugal, targeting *Blastocystis* sp., *Balantioides coli*, *Cryptosporidium* spp., *Encephalitozoon* spp., and *Enterocytozoon bieneusi*. Fecal samples from 71 ducks were analyzed using PCR and sequencing techniques. The results revealed a 2.82% occurrence of *Blastocystis* sp. subtype 7 and *Cryptosporidium baileyi*, marking the first molecular detection of these pathogens in wild ducks in Portugal. While previous studies have documented these parasites in *Anas* spp. in other regions, this study contributes novel data specific to the Portuguese context. No evidence of *Balantioides coli*, *Encephalitozoon* spp. or *Enterocytozoon bieneusi* was found. These findings highlight the potential role of migratory ducks as vectors for zoonotic protozoa, emphasizing the need for enhanced surveillance of avian populations to mitigate cross-species transmission risks. Further research is warranted to understand the global public health implications associated with migratory waterfowl.

## 1. Introduction

Diarrheal diseases caused by enteric parasites are among the top 10 causes of death according to the World Health Organization (WHO) [1], highlighting their significance in relation to both public and veterinary health. Some of the most important enteric parasites worldwide include *Blastocystis* sp. (Stramenopiles), *Balantioides coli* (Ciliophora), and *Cryptosporidium* spp. (Apicomplexa), as well as the *Encephalitozoon* spp. and *Enterocytozoon bieneusi* (Microsporidia) [2,3,4,5].

These parasites are capable of infecting a broad range of hosts, including humans [6,7,8,9]. The primary route of transmission is the fecal–oral route, through the accidental ingestion of their infective stages (cysts, oocysts, or spores) [10,11,12]. Transmission can occur directly through contact with infected humans or animals, or indirectly through consumption of contaminated water or food sources [12,13,14,15]. Migrating ducks, namely *Anas acuta*, *A. platyrhynchos* and *A. crecca*, have previously been reported in different countries, including the USA, Iran, Cyprus, Poland, Spain, New Zealand, Algeria and Brazil, to harbor and shed diarrheagenic parasite infections, including *Blastocystis* sp., *Cryptosporidium* spp., and microsporidia [16,17,18,19,20,21,22,23,24,25].

*Anas acuta*, *Anas platyrhynchos*, and *Anas crecca* have distinct migratory routes shaped by seasonal changes. Northern Pintails (*Anas acuta*) leave their northern breeding grounds in North America, Europe, and Asia in autumn. Those from northwest Europe, including Iceland and Scandinavia, migrate southwest along the Atlantic coast to winter in western and southern Europe, while Pintails from Siberia and central Asia head to the Mediterranean, Black Sea, and Caspian Sea, with some reaching India and tropical West Africa. Their return migration begins in March and continues into May [26]. Mallards (*Anas platyrhynchos*) begin their autumn migration in August, with birds from northwest Russia and the Baltic states wintering in western Europe, while those from central and eastern Europe move to the Mediterranean and Black Sea. Mallards from central Russia, western Siberia, and Kazakhstan migrate southwest, reaching as far as Iran and Afghanistan. The return migration for Mallards intensifies in March and continues into May [26]. Common Teals (*Anas crecca*) start migrating southwest in July, with Siberian populations traveling to the eastern Mediterranean and Black Sea, and some following the Nile to East Africa. Their spring migration intensifies as they return northeast to their breeding areas. Together, these species demonstrate the diverse migratory behaviors of ducks as they adapt to environmental changes in their habitats [26]. *Balantioides coli* is the only ciliate known to infect humans, and it is also the largest parasitic protozoan capable of doing so [5]. While pigs are the primary reservoir for this parasite, other animals, including camels, cattle, donkeys, sheep, and goats, have been suggested as potential reservoirs for human infections [8]. *Balantioides coli* inhabits the intestinal tract of the host, where infection may remain asymptomatic or be present with clinical manifestations ranging from mild intestinal disturbances to severe, life-threatening colitis [8]. Molecular characterization of *B. coli* is achieved through the analysis of the ITS1–5.8S rRNA–ITS2 sequences. This analysis has enabled the identification of two genetic variants (A and B) and five subvariants (A0, A1, A2, B0, and B1) across isolates from humans, pigs, ostriches, and non-human primates [27]. Although a limited number of outbreaks in humans have been reported, there is scarce information regarding this parasite in migratory ducks [8]. Nonetheless, *B. coli* has been documented in other wild avian species, such as greater and lesser rheas, as well as ostriches [28,29].

The stramenopile *Blastocystis* is one of the most common gastrointestinal parasites and is highly polymorphic, with uncertain pathogenicity [30]. It is present in the human gut, with an estimated colonization rate of between one and two billion worldwide [31]. This parasite is divided into different subtypes (STs), with a total of 42 legitimate STs identified so far, 17 of which are found in humans (ST1–ST10, ST12, ST14, ST16, ST23, ST26, ST35, and ST41) [32]. Notably, 90% of human infections are represented by ST1–ST4 [33]. Some STs are considered to originate from animals, such as cattle (ST10 and ST14), birds (ST6 and ST7), pigs (ST5), and non-human primates (ST8) [32]. In wild ducks, potentially zoonotic STs have been previously reported, such as *Blastocystis* sp. ST7 in *Anas platyrhynchos* and ST7, ST10, ST27, ST28 in *A. querquedula* [25].

*Cryptosporidium* is considered the second leading cause of diarrhea globally, after rotavirus, and significantly contributes to child mortality and morbidity under 5 years old worldwide [34]. To date, at least 51 species of *Cryptosporidium* have been identified, along with approximately 120 genotypes [35,36,37]. Among these, over 20 species have been detected in humans, with *C. parvum* and *C. hominis* being the most common, responsible for approximately 95% of human infections [38]. Studies on wild ducks, specifically those of the *Anas* genus, remain limited. The few reports of *Cryptosporidium* in these ducks include *C. parvum*, *C. bailey*, and *C. proventriculi* (formerly *Cryptosporidium* avian genotype III) [19,39,40].

Miscrosporidia, particularly species from the genus *Encephalitozoon* and *Enterocytozoon bieneusi* (the only member of the *Enterocytozoon* genus capable of infecting humans), are the most common causes of microsporidiosis worldwide [41]. Among the *Encephalitozoon* species, *E. intestinalis* is the most frequently found in humans [15]. *E. cuniculi* is primarily associated with rabbits, while *E. hellem* is one of the most common species found in birds, including wild ducks, suggesting that birds may serve as the primary hosts [24,42,43,44,45]. The described *E. cuniculi* and *E. hellem* can be divided into different genotypes based on differences in the ITS region. *Encephalitozoon cuniculi* has thus been classified into genotypes I, II, III, and IV, while *E. hellem* is divided into genotypes 1, 2, and 3 based on the ITS sequence variations [46]. For now, there is no evidence of intraspecific polymorphisms in the ITS gene of *E. intestinalis* in any study [47]. While genotyping aids in identifying strains with a certain host preference, recent studies have shown that these strains do not exhibit strict host specificity [15]. Consequently, humans can be infected by any of the four *E. cuniculi* strains, as well as by *E. hellem* or *E. intestinalis* [15].

Among microsporidia, *Enterocytozoon bieneusi* is the most prevalent cause of human microsporidiosis, accounting for approximately 90% of cases [48]. This microsporidium has over 500 genotypes, and the most recent studies have divided them into 15 groups [49]. Of these, group 1 is the largest, followed by group 2, both of which are of major zoonotic importance, as they include the highest number of zoonotic genotypes [50]. Groups 3–15 are typically considered to be host-specific, suggesting lower significance in terms of the zoonotic potential [49]. Based on a recent review of migrating Anatidae, there are no records of *E. bieneusi* presence in migratory ducks, although it has been recorded in other migratory bird species [16].

Migratory waterfowl can act as mechanical vectors for spreading enteric parasites, picking up infections from their habitats and transmitting them to the environment, including water sources, and to other animals. While prior research has documented the presence of *Blastocystis* sp., *Cryptosporidium* spp., *Encephalitozoon* spp. and *Enterocytozoon bieneusi* in migratory ducks, our study represents the first molecular detection and characterization of *Blastocystis* sp. ST7 and *Cryptosporidium baileyi* in wild migratory ducks captured in Portugal.

## 2. Materials and Methods

### 2.1. Sampling

**Figure 1:**
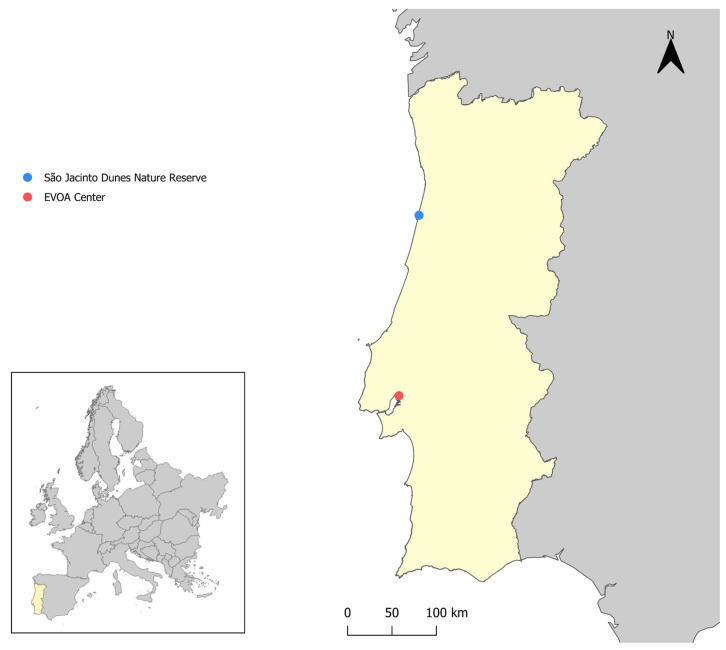
Geographic illustration of the sample collection sites in this study.

A total of 71 fecal samples were collected from ducks using cotton swabs at the cloaca and stored at −20 °C. The sampled species included 37 Mallard (*Anas platyrhynchos*), 25 Pintail (*Anas acuta*), and 10 Teal (*Anas crecca*). The ducks were captured in São Jacinto Dunes Nature Reserve (Aveiro) and at EVOA (Tagus River Estuary Nature Reserve, Vila Franca de Xira) (Figure 1). These areas are known for high concentrations of ducks and extensive ecological studies. The ducks were marked with nasal saddles for tracking [51], and their capture was authorized under permit number 40/2021 from the Instituto da Conservação da Natureza e das Florestas (ICNF), Portugal. The sampling performed in this study was previously used in a coronavirus research project [52].

### 2.2. DNA Extraction

DNA extraction from the fecal swabs involved thorough mixing by vortexing in 500 µL of phosphate-buffered saline (PBS) with a pH of 7.2. The mixture was then centrifuged at 8000× *g* for 5 min. After centrifugation, 140 μL of the supernatant was used for DNA extraction and purification with the QIAamp DNA Mini Kit (Qiagen, Hilden, Germany), following the manufacturer’s instructions. The extraction was automated using the QIAcube^®^ platform (Qiagen). The purified DNA was then stored at −80 °C in nuclease-free water.

### 2.3. Molecular Detection of Enteric Parasites

The molecular detection of *Blastocystis* sp. was performed using real-time PCR (qPCR) with the primer set BL18SPPF1/BL18SR2PP, targeting the small ribosomal subunit (SSU) rRNA gene to amplify an approximately 300 bp fragment, as previously described [53]. For *B. coli*, standard PCR was used, targeting the ITS1–5.8S rRNA–ITS2 region and the final 117 bp of the SSU rRNA gene. The primer set B5D/B5RC amplified a 400 bp product, following the method described in [54]. The detection of *Cryptosporidium* spp. was achieved by nested PCR, targeting the SSU rRNA gene. The first round used the primer set CR-P1/CRP2, followed by CR-P3/CPB-DIAGR in the second round, yielding a 587 bp fragment, as described in [55]. For *Encephalitozoon* spp., nested PCR was employed, targeting the SSU rRNA genomic region. The first round used the PMicF/PMicR primers, amplifying a 779 bp fragment, and the second round used EncepF/EncepR, generating a 440 bp product, following the method described in [56]. Finally, *Enterocytozoon bieneusi* detection was performed by nested PCR targeting the ITS region and flanking small and large subunits of the rRNA gene. The first round used the primer pair EBITS3/EBITS4, amplifying a 435 bp fragment, while the second round employed EBITS1/EBITS2.4, producing a 390 bp fragment, as described in [57].

All the molecular assays were performed by including positive and negative controls on each reaction.

Except for the positive qPCR results, which were directly purified and sequenced, DNA fragments amplified by standard and nested PCR were separated using electrophoresis on 1.5% agarose gels stained with Xpert Green Safe DNA gel dye (GRiSP^®^, Porto, Portugal). Electrophoresis was performed at a constant voltage of 120 V for 30 min, and the DNA bands were visualized under UV light.

### 2.4. Sanger Sequencing and Phylogenetic Analysis

The qPCR-positive samples for *Blastocystis* sp. were purified and sequenced by Genoscreen (Lille, France). For the other enteric parasites, standard and nested PCR amplicons matching the expected size were purified using the GRS PCR and Gel Band Purification Kit (GRiSP^®^, Porto, Portugal) and subsequently sequenced via the Sanger dideoxy sequencing method by the company STABvida (Monte da Caparica, Portugal). The nucleotide sequences were processed, aligned, and analyzed using BioEdit Sequence Alignment Editor version 7.2.5 [58]. The consensus sequences were then compared to those in the NCBI GenBank database through the Nucleotide Basic Local Alignment Search Tool (BLASTN). For further analysis, MEGAX software version 10.2.6 was used [59]. To assess the phylogenetic relationships between *Blastocystis* sp. and *Cryptosporidium* spp., a maximum-likelihood tree using SSU rRNA was created with MEGA X [59]. The construction of the tree utilized substitution rates calculated through the Tamura-3-parameter model [60], along with a gamma distribution that included invariant sites (+G). Bootstrapping with 1000 replicates was performed to evaluate the support for the clades. The sequences from this study have been submitted to the GenBank database and assigned unique accession numbers: PQ312881, PQ312882 (*Blastocystis* sp.), PQ312883 and PQ312884 (*Cryptosporidium baileyi*).

### 2.5. Statistical Analysis

The prevalence of enteric parasites in the duck population studied was determined by calculating the ratio of positive samples to the total number of samples analyzed, with a 95% confidence interval (95% CI). Data processing and the initial analysis were carried out using Microsoft Excel^®^ for Microsoft 365 MSO (version 2312 Build 16.0.17126.20132, 64-bit).

## 3. Results

### 3.1. Occurrence of Blastocystis *sp.*, Balantioides coli, Cryptosporidium *spp.* and Microsporidia

A total of two samples tested positive for *Blastocystis* sp., scoring an overall occurrence of 2.82% (2/71; 95% confidence interval [CI]: 0.34–9.81). Additionally, two samples tested positive for *Cryptosporidium* spp., also scoring an overall occurrence of 2.82% (2/71; 95% confidence interval [CI]: 0.34–9.81). None of the samples of *A. crecca* tested positive for any enteric parasites, and none of the 71 samples tested positive for *B. coli* or microsporidia. A more detailed description of the occurrences per species and parasite is shown in Table 1.

### 3.2. Molecular Characterization of Blastocystis *sp.* and Cryptosporidium *spp.*

For *Blastocystis* sp., BLAST analysis showed that the sequences PQ312881 and PQ312882 from this study exhibited 100% identity with the sequence MW093216 from a chicken in Turkey and 99.57% identity with the sequence MW888502 from a fox in China, respectively. The *Cryptosporidium* spp. sequences PQ312883 and PQ312884 both shared 100% identity with the *Cryptosporidium baileyi* sequence KT151528 from a chicken in Iraq.

**Figure 2:**
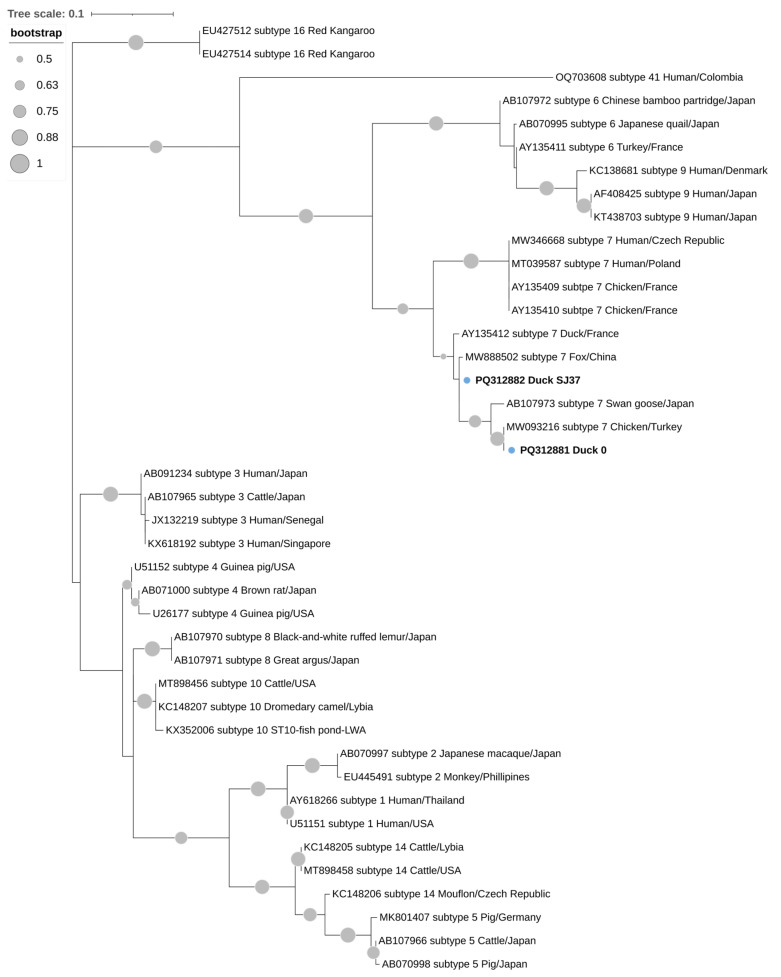
Phylogenetic relationships of the (SSU-rRNA) gene of *Blastocystis* sp. isolates obtained in this study (in bold) and reference sequences retrieved from GenBank, along with their respective accession numbers, STs, hosts, and countries of origin. The phylogenetic analysis was conducted using MEGA X software with the maximum likelihood method, applying the Tamura-3-parameter model to calculate the genetic distances. The proportion of replicate trees in which the related taxa grouped together during the bootstrap test (1000 replicates) is indicated beside the branches. Bootstrap values under 50% are not displayed. The phylogenetic tree was edited using the Interactive Tree of Life (iTOL).

**Figure 3:**
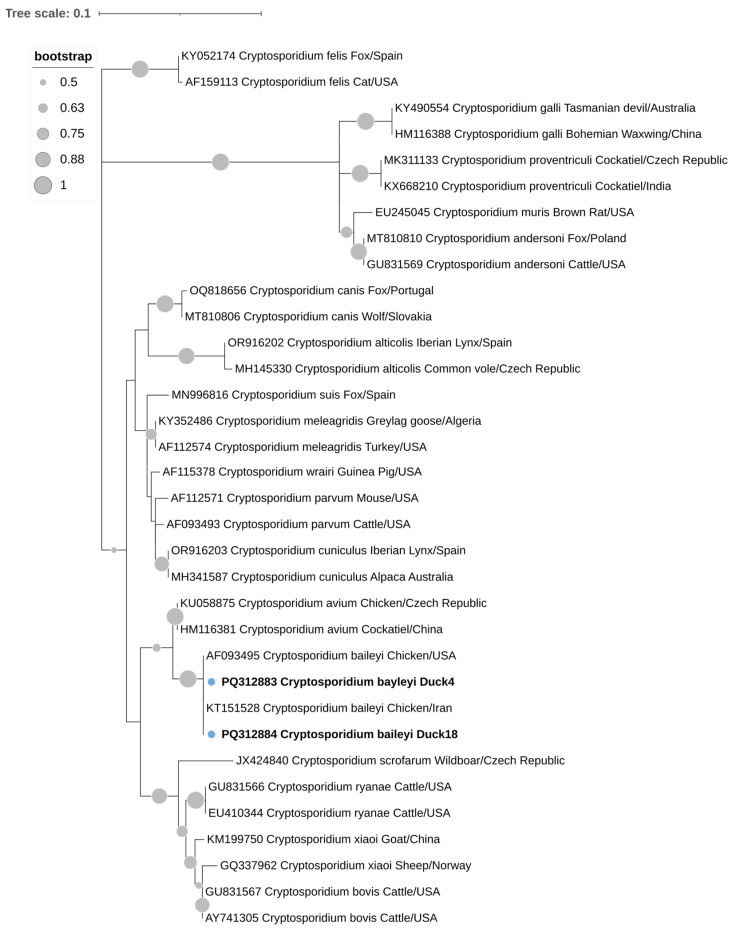
Phylogenetic relationships of the (SSU-rRNA) gene of *Cryptosporidium* spp. isolates obtained in this study (indicated in bold) and reference sequences retrieved from GenBank, along with their respective accession numbers, species, hosts, and countries of origin. The phylogenetic analysis was conducted using MEGA X software with the maximum likelihood method, applying the Tamura-3-parameter model to calculate the genetic distances. The proportion of replicate trees in which the related taxa grouped together during the bootstrap test (1000 replicates) is indicated beside the branches. Bootstrap values under 50% are not shown. The phylogenetic tree was edited using the Interactive Tree of Life (iTOL).

The analysis of the SSU rRNA regions of the four positive samples confirmed the presence of *Blastocystis* sp. ST7 and *Cryptosporidium baileyi*, as shown in Figure 2 and Figure 3, respectively, where they are grouped together with the corresponding reference sequences in well-defined clusters.

## 4. Discussion

This study is the first to investigate wild ducks (*A. acuta*, *A. platyrhynchos* and *A. crecca*) in Portugal concerning the molecular identification and characterization of several enteric parasites, namely *Blastocystis* sp., *Balantioides coli*, *Cryptosporidium* spp., *Encephalitozoon* spp., and *Enterocytozoon bieneusi*. The results highlight the presence of enteric parasites in two of the three species tested, namely *A. acuta* and *A. platyrhynchos*. *Blastocystis* sp. ST7 was found in both *A. acuta* and *A. platyrhynchos*, while *Cryptosporidium baileyi* was found exclusively in *A. acuta*.

*Blastocystis* is a common microeukaryote found in human and animal feces, with ST1 to ST4 being the most prevalent, showing a 90% infection rate. Its pathogenicity regarding humans is still under debate, but some STs, like ST7, are linked to negative gut health effects [61]. ST7 can disrupt beneficial gut bacteria, such as *Bifidobacterium longum* and *Lactobacillus brevis*, and produce proteases that damage the intestinal barrier, potentially increasing vulnerability to other pathogens [61,62,63,64,65]. The immune response to ST7 may also cause inflammation, though further research is needed to clarify its health implications [63,66,67,68].

The identification of *Blastocystis* sp. ST7 in *A. platyrhynchos* raises concerns due to the widespread distribution of this dabbling duck species [69]. Mallards interact with both wild and domestic birds, creating opportunities for cross-species transmission of enteric pathogens. The detection of *Blastocystis* in *A. acuta* and *A. platyrhynchos* of 4.17% and 2.7%, respectively, suggests that migratory waterfowl could be vectors for the global spread of these protozoa and other zoonotic parasites. Since *Blastocystis* sp. ST7 can colonize various host species, this finding emphasizes the need for increased surveillance in avian populations to prevent potential zoonotic spillovers.

Previous studies on wild birds are very limited but suggest that migratory ducks may harbor a variety of *Blastocystis* STs. For example, a study conducted in Brazil on captive and wild birds identified ST7, ST10, ST27, and ST28 in *A. platyrhynchos* and *A. querquedula*, with an occurrence rate of 66.7% [25]. Although this prevalence is substantially higher than the 2.82% detected in this study, discrepancies in the sample size, ecological conditions, and geographic factors warrant careful interpretation. Importantly, the detection of ST7 in Portugal expands its known geographical distribution and host range, reinforcing the potential risk of global dissemination via migratory waterfowl.

In contrast to *Blastocystis*, *C. baileyi* was detected exclusively in *A. acuta*, with an occurrence rate of 8.33%. While *C. baileyi* is primarily an avian parasite, members of the *Cryptosporidium* genus, including *C. baileyi*, have zoonotic potential and have been documented in human infections, although the risk and recurrence are low [70,71,72]. *Cryptosporidium* is particularly concerning due to its ability to cause gastrointestinal illness, especially in immunocompromised individuals, making it a public health concern.

Although *Cryptosporidium baileyi* infection in humans is rare and poses a low risk, its detection in migratory ducks highlights the potential threat these birds present as vectors for the transmission of cryptosporidiosis, especially in areas where wildlife, livestock, and human populations overlap. *Cryptosporidium baileyi* is the most common species found in domestic poultry and wild birds and is a significant cause of respiratory and intestinal infections, leading to morbidity and mortality worldwide due to respiratory disorders, which can result in financial losses [73,74]. Given their vast migration distances and diverse habitats, these ducks can facilitate the wide distribution of *Cryptosporidium* spp., contributing to the transmission dynamics of cryptosporidiosis across regions. Comparisons with other studies reveal variability in the detection rates of *Cryptosporidium* spp. in migratory ducks. For example, *C. baileyi* has been reported in *Anas* species with a prevalence of 3.22% in Algeria, 11.5% in Japan, and up to 47.7% in Mexico [17,23,40]. The lower occurrence in this study may reflect geographic or ecological differences, as well as sample sizes.

This study found no evidence of *B. coli*, *Encephalitozoon* spp. *E. bieneusi* in the examined duck populations. To the best of our knowledge, there are no previous reports in the literature on the presence of both *B. coli* and *E. bieneusi* in wild migratory ducks. This absence highlights the need for further investigation into the potential role of waterfowl in the transmission of these organisms, particularly considering their zoonotic potential. In contrast, *Encephalitozoon* spp. have been detected in previous studies, with a notable occurrence of *E. hellem* at 18% in *A. platyrhynchos* from Poland [24]. This discrepancy suggests that while some enteric pathogens may be less prevalent in migratory ducks, others, such as *Encephalitozoon* spp., need closer attention due to their demonstrated presence in avian hosts and potential zoonotic implications.

## 5. Conclusions

This study provides the first molecular evidence of *Blastocystis* sp. ST7 and *Cryptosporidium baileyi* in migratory ducks captured in Portugal, shedding light on the complex role of migratory waterfowl in the dissemination of zoonotic pathogens. The identification of these enteric parasites in *A. acuta* and *A. platyrhynchos* emphasizes the need for enhanced monitoring of avian populations, particularly migratory species that traverse multiple ecological zones and interact with various wildlife, livestock, and human populations. Given the global distribution of these ducks and their potential to carry zoonotic pathogens, further research is needed to assess the public health risks associated with migratory waterfowl and to develop strategies to mitigate the transmission of zoonotic diseases.

## Figures and Tables

**Table 1:** Occurrence of *Blastocystis* sp., *Balantioides coli*, *Cryptosporidium* spp., and microsporidia in the duck species analyzed in this study. The table presents the number of samples tested and the number of positive cases (%, 95% confidence interval [CI]) for each parasite across the duck species *Anas platyrhynchos*, *Anas acuta*, and *Anas crecca*, as well as the total occurrence for each parasite.

Duck Species	No. of Sample Tested	No. of Positives for *Blastocystis* sp. (%, 95% CI)	No. of Positives for *Balantioides coli* (%; 95% CI)	No. of Positives for *Cryptosporidium* spp. (%; 95% CI)	No. of Positives for Microsporidia (%; 95% CI)
*Anas* *platyrhynchos*	37	1 ^E^ (2.7%; 95% CI: [0.07–14.16])	0	0	**0**
*Anas acuta*	24	1 ^E^ (4.17%; 95% CI: [0.11–21.12])	0	2 ^E, S^ (8.33; 95% CI: [1.03–27.0])	**0**
*Anas crecca*	10	0	0	0	**0**
Total	71	2 (2.82; 95% CI: [0.34–9.81])	0	2 (2.82; 95% CI: [0.34–9.81])	**0**

^E^ Positive samples from EVOA Center. ^S^ Positive samples from São Jacinto Dunes Nature Reserve.

## Data Availability

The data presented in this study are available on request from the corresponding author.
